# Preparing for patient partnership: A scoping review of patient partner engagement and evaluation in research

**DOI:** 10.1111/hex.13040

**Published:** 2020-03-10

**Authors:** Marissa Bird, Carley Ouellette, Carly Whitmore, Lin Li, Kalpana Nair, Michael H. McGillion, Jennifer Yost, Laura Banfield, Elaine Campbell, Sandra L. Carroll

**Affiliations:** ^1^ Faculty of Health Sciences School of Nursing McMaster University Hamilton ON Canada; ^2^ Population Health Research Institute Hamilton ON Canada; ^3^ M. Louise Fitzpatrick College of Nursing Villanova University Villanova PA USA; ^4^ Health Sciences Library McMaster University Hamilton ON Canada; ^5^ Patient Partner McMaster University Hamilton ON Canada

**Keywords:** evaluation studies, patient engagement, patient oriented research, patient participation, patient partners, scoping review

## Abstract

**Background:**

Realizing patient partnership in research requires a shift from patient participation in ancillary roles to engagement as contributing members of research teams. While engaging patient partners is often discussed, impact is rarely measured.

**Objective:**

Our primary aim was to conduct a scoping review of the impact of patient partnership on research outcomes. The secondary aim was to describe barriers and facilitators to realizing effective partnerships.

**Search Strategy:**

A comprehensive bibliographic search was undertaken in EBSCO CINAHL, and Embase, MEDLINE and PsycINFO via Ovid. Reference lists of included articles were hand‐searched.

**Inclusion Criteria:**

Included studies were: (a) related to health care; (b) involved patients or proxies in the research process; and (c) reported results related to impact/evaluation of patient partnership on research outcomes.

**Data Extraction and Synthesis:**

Data were extracted from 14 studies meeting inclusion criteria using a narrative synthesis approach.

**Main Results:**

Patient partners were involved in a range of research activities. Results highlight critical barriers and facilitators for researchers seeking to undertake patient partnerships to be aware of, such as power imbalances between patient partners and researchers, as well as valuing of patient partner roles.

**Discussion:**

Addressing power dynamics in patient partner‐researcher relationships and mitigating risks to patient partners through inclusive recruitment and training strategies may contribute towards effective engagement. Further guidance is needed to address evaluation strategies for patient partnerships across the continuum of patient partner involvement in research.

**Conclusions:**

Research teams can employ preparation strategies outlined in this review to support patient partnerships in their work.

## 1 INTRODUCTION

Including patient partners in research holds promise for targeting patient‐important research questions, creating meaningful change in patient outcomes and health systems, and realigning both research processes and outcomes to be patient‐centred.^1^ Internationally, efforts to grow inclusion of patient representatives on research teams have been driven by funding bodies, government and calls to action by patient communities.^2^, ^3^, ^4^ Patient partnership represents a growing niche in the broader field of patient engagement, whereby patients take on a more collaborative than contributory role in the research process. Consistent with definitions from the Patient‐Centered Outcomes Research Institute (PCORI)^5^ and the Strategy for Patient Oriented Research Patient Engagement Framework (SPOR),^6^ the term ‘patient partners’ within this manuscript is intended to include patients and their proxies (ie family members or caregivers) who are directly involved as members of the research team, as opposed to being consenting research participants. In this study, this definition was operationalized by only including patient partners who were not consented as study participants, but members of the research team. On the continuum of patient engagement practices described by Forsythe and colleagues,^4^ as well as the Levels of Patient and Researcher Engagement in Health Research proposed by Manafo et al^7^ patient partnerships are typically those that involve collaboration, shared leadership practices and patient partner embeddedness in research teams as co‐investigators. In contrast to broad engagement practices which commonly involve unidirectional input from patients to researchers via solicitation of viewpoints or experiences to inform the research agenda, patient partner relationships within research teams are characterized by bidirectional information flow and active decision making and collaboration.^4^

Historical efforts aimed at involving patients in research began with broad engagement of various stakeholder groups. For example, within the Canadian health‐care system, the Canadian Institutes of Health Research (CIHR) 2014/15‐2018/19 Strategic Plan, *Health Research Roadmap II (HRR‐II)*, focuses on mobilizing health research for transformation and optimal impact on the health of Canadians.^8^ To meet this challenge, the *HRR‐II* calls for scientific leaders to employ a highly networked approach, inclusive of multiple stakeholder groups, in order to transcend traditional strategic alliances for health research. A key priority for such collective action is partnership with citizens, patients and caregivers with a view to effectively accelerate innovation, and ultimately create better health and health care. Similar initiatives have been fully launched outside of Canada including INVOLVE, funded by the National Health Service in the United Kingdom, and the Patient‐Centered Outcomes Research Institute (PCORI) in the United States.

A primary driver in the field of patient engagement is active collaboration with patients on research teams – including family, caregivers and friends – in governance, priority setting, conduct of research, and knowledge translation.^9^ However, many researchers continue to struggle with how to operationalize research partnerships with patients, both realistically and effectively. We propose that partnerships require a shift in focus from engaging research participants in ancillary roles to more active roles, wherein patients engage as collaborative team members throughout the research process. While patient partnerships are often discussed, their impacts are rarely measured.^10^ Previous systematic reviews^2^, ^3^, ^11^, ^12^ on the involvement and engagement of patients in research have not differentiated patient partners from consenting research participants who are engaged in research. A knowledge gap exists related to whether patient partnerships – as a subset of patient engagement – have an impact on research outcomes.

## 2 METHODS

### 2.1 Study aims

The primary aims of this work were as follows: (a) to identify the body of research where patients were included as patient partners in the research process; and (b) to determine whether the impact of patient partnership was examined and, if so, what impact the partnership had on research outcomes. The secondary aim was to describe specific partnership methods, barriers and facilitators across included studies in order to make practical recommendations about operationalizing patient partnerships.

### 2.2 Guiding framework

The scoping review was guided by the Arksey and O'Malley Scoping Review Framework^13^ which lends structure for identifying the research question and relevant studies, selecting studies, charting the data, and collating, summarizing and reporting results.

### 2.3 Research question

Within the existing body of health research literature, how is the impact of patient partnership being examined and, when examined, what impact does partnership have on research outcomes?

### 2.4 Search process

The search strategy was developed with an experienced information scientist in consultation with the research team. The search was undertaken in Ebsco CINAHL, and EMBASE, MEDLINE and PsycINFO via Ovid. No limits for language, date or publication type were applied. Using a combination of keywords and database‐specific subject headings, the concept of community‐engaged/patient partnership research (encompassing community‐institution relationships and patient or consumer participation) was constructed as a concept for this search. This concept was then combined with language describing health services research or research design to form the basis of the search strategy. All databases were searched from database inception to October 2019. For a sample MEDLINE search, see Appendix 1 (other search strategies are available on request). Once duplicates were removed, citations were uploaded to a web‐based program (Distiller SR)^14^ and the title and abstract of each citation were screened by one reviewer. In addition, reference lists of included articles were hand‐searched for possible additional articles.

### 2.5 Inclusion criteria

The population of interest was defined as patients or their proxies including informal caregivers, family and/or friends who were considered patient partners during any stage of health research. Only studies where patient partners had not signed consent were included in this review as we interpreted the practice of consent to imply a research participant role versus a partner in the research process. All quantitative, qualitative or mixed methods peer‐reviewed research articles from any health‐care setting were included. Included articles described an evaluation of the impact of patient partnerships on research outcomes using validated tools for quantitative studies, or patient partner interviews or focus groups for qualitative studies. This applied examination of the impact of patient partnerships necessarily excluded studies where the objective was to develop a measure or tool to evaluate patient partnership, for example (see Appendix 2 for Screening Criteria).

### 2.6 Selection of studies

At the initial screening level, both titles and abstracts of citations were screened by a single reviewer. Full‐text screening was conducted independently by two reviewers, with agreement necessary for inclusion. Disagreements were reconciled through discussion prior to exclusion of studies.

### 2.7 Data extraction

**Figure 1:**
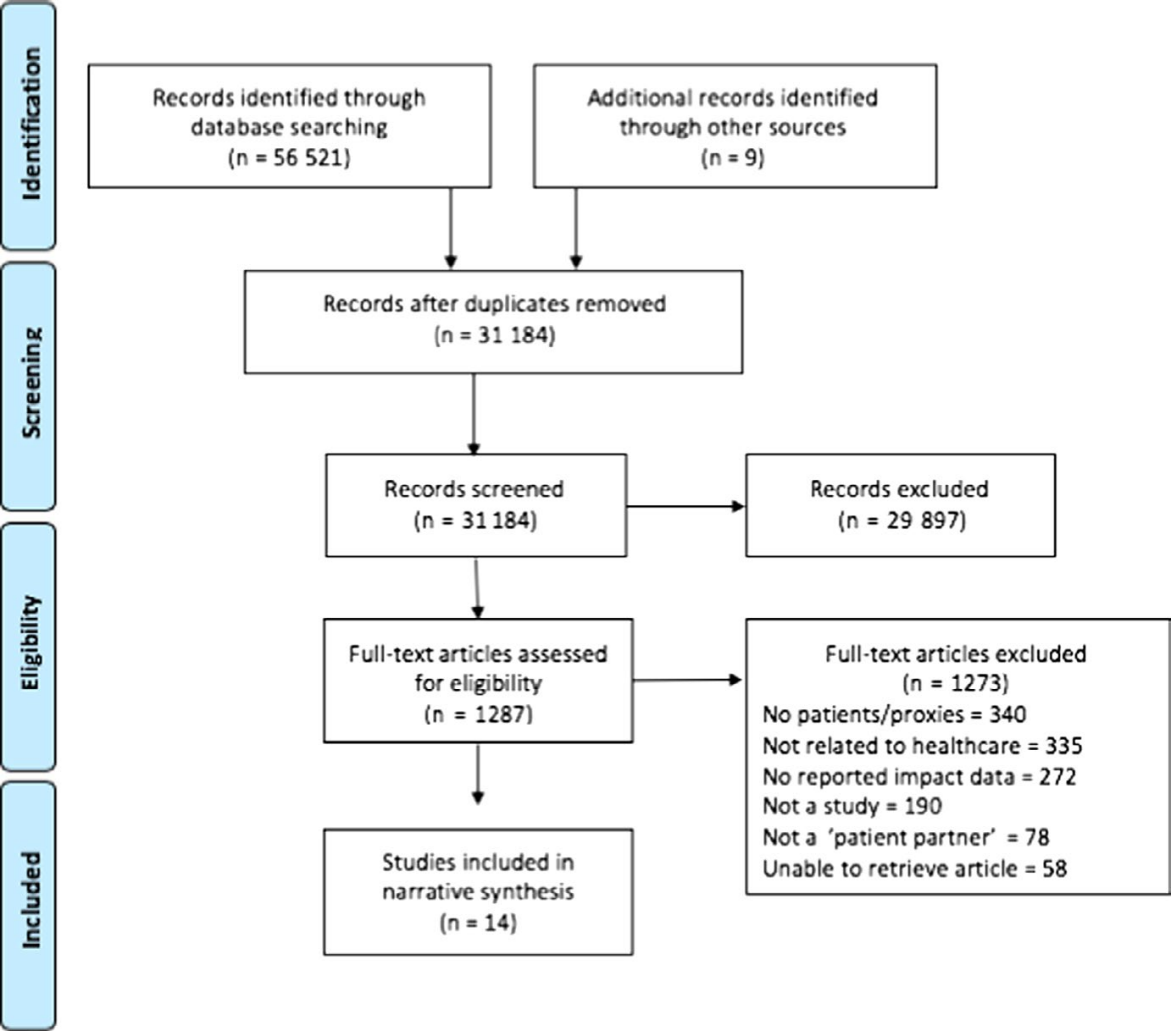
PRISMA flow diagram. From: Moher et al^53^

Data extraction from included studies was independently completed by two reviewers. All disagreements were resolved through consensus between reviewers. Study characteristics were extracted, as well as process for patient partner involvement, patient partner role/contribution, barriers and facilitators to partnership, approach to evaluation, and ‘stage’ of patient engagement (Manafo et al^7^). Data were extracted using a standardized data extraction form. See Figure 1 for flow of studies through the phases of selection, data extraction and synthesis, according to Preferred Reporting Items for Systematic Reviews and Meta‐Analyses (PRISMA) guidelines.^15^

### 2.8 Data analyses

Results were summarized using a narrative synthesis process consistent with Arksey and O’Malley's Framework.^13^ Our approach to narrative synthesis followed guidance provided by Popay and colleagues^16^ and included a descriptive summary of study characteristics (including country of origin, research method, diagnostic focus and patient partnership method), and an exploration of relationships between studies' reported findings. Study findings were grouped into categories related to perceived patient partner contribution, as well as barriers/facilitators to partnership. Each included study was also assigned a level of patient engagement as proposed by Manafo et al^7^ in order to depict variations in the operationalization of patient partnerships across studies. Findings were described and synthesized across studies.

## 3 RESULTS

The search yielded 31 184 unique citations which included 31 175 from the database review and nine studies from a previously published systematic review.^12^^.^ After title and abstract screening, 29 897 citations were excluded, leaving 1287 for full‐text review. After full‐text screening, an additional 1273 articles were excluded, leaving a total of 14 included studies in this review.

All 14 of the included studies utilized qualitative data collection and analysis techniques to evaluate the impact of patient partner engagement on research outcomes.^17^, ^18^, ^19^, ^20^, ^21^, ^22^, ^23^, ^24^, ^25^, ^26^, ^27^, ^28^, ^29^, ^30^ Table 1 summarizes the key study characteristics. Eight studies originated from the United Kingdom, 18, 19, 20, 22, 23, 24, 25, 26 one from Canada,^21^ one from Sweden,^27^ and four from the United States of America.^17^, ^28^, ^29^, ^30^ The included studies used a combination of qualitative data collection methods, including interviews (n = 8),^17^, ^18^, ^19^, ^20^, ^21^, ^24^, ^28^, ^30^ focus groups (n = 8),^17^, ^18^, ^20^, ^22^, ^23^, ^24^, ^25^, ^26^ questionnaires (n = 5),^22^, ^24^, ^27^, ^28^, ^29^ reflections (n = 1)^27^ and observations (n = 7).^18^, ^21^, ^25^, ^26^, ^27^, ^29^, ^30^ The focus of five of the studies was on chronic conditions, including musculoskeletal conditions,^26^ diabetes,^23^ stroke,^20^ Parkinson's disease^27^ and asthma.^30^ Three of the studies examined populations with cancer,^18^, ^19^, ^22^ two were in varied populations,^25^, ^28^ one focused on the primary care setting,^29^ and another involve street‐involved youth.^21^ Additionally, one study focused on sexuality in older adults,^24^ and one study evaluated patient partnerships with adults with developmental disabilities.^17^

**Table 1:** Patient partnership characteristics in included Studies

Author, (year), Country	Study focus	Health condition/topic	Process for patient partner involvement	Patient partner role or contribution	Level of engagement^7^	Impact evaluator
Boaz et al, (2016), UK	To understand the roles of patients and carers who took part in one of four co‐designed quality improvement interventions related to clinical pathways for intensive care units and lung cancer in two NHS trusts. Process evaluation of implementation process.	Intensive Care Units; lung cancer	Evaluation included 155 hours of observation from training sessions, events, co‐design meetings, advisory and core group meetings, and 30 interviews with patients, carers, staff, and facilitators, and 2 group interviews with patients and carers.	Participants provided insights into the types of roles that patients and carers took on during the implementation process. This included sharing of experience, offering suggestions for change, and implementing possible solutions. Study highlighted that impact was on a smaller scale; however, partners provided ideas for change and possible solutions as well as motivated staff for possible changes.	Consult, Involve	Unclear
Brown et al, (2018), UK	To reflect on the public involvement practices that underpinned the Older People's Understanding of Sexuality (OPUS) project. Project focus on intimacy and sexuality in care homes. Reflective commentaries on the team approach to patient involvement.	Sexuality and intimacy	Two community representatives were invited to participate in all aspects of the research that did not require additional training (eg recruitment, data collection, and data analysis). Community representatives were included in all study correspondence and meeting information, and took part in study discussions.	Community representatives were included in discussion and decision making regarding plans for recruitment, thematic analysis, changes to study plan, broader dissemination of study findings, and future grant work. Stemming from a presentation at a half‐day conference by community representatives, a sense of authenticity of study results was described.	Collaborate	Research team
Froggatt et al, (2015), UK	To understand the experiences, benefits and challenges for research partners (patient and public with cancer) who took part in activities related to cancer research.	Cancer	Research partners with a known diagnosis of cancer were invited to take part in an interview to describe their experience within the research projects.	Participants contributed to the inclusion of a lay perspective for the research, offered practical viewpoints on the research, and acquired new knowledge and skills, confidence and personal support for their illness experience.	Participate, Consult	Research team and patient partners
Howe et al, (2017), UK	To examine patient and public involvement in the RAPPORT project. Comparisons are drawn between RAPPORT conclusions and the experiences of the authors. Examined patient and public engagement developed over time, the challenges and barriers, changes made because of input from patient partners, and evidence related to changes and lessons learned.	Varied	Patient partner involvement included membership on the research team, the research advisory group and reference group for specific topics. Data sources included project documentation, meeting minutes, feedback after meeting activities, resources offered to patient partners and structured feedback of two formal, independently run, reflective meetings.	All patient partner representatives and researchers expressed an increase in their confidence in all described roles over time.	Co‐applicants: Involve, Support, Collaborate Advisory Group Members: Collaborate, Involve Patient Groups: Collaborate, Involve	External, independent
Hyde et al, (2017), UK	To describe the process and impact of patient and public involvement and engagement in a systematic review of factors affecting shared decision making around prescribing analgesia for musculoskeletal pain in primary care consultations.	Musculoskeletal conditions	Five members of a patient Research User Group (RUG) collaborated with researchers in the review process. This was facilitated by a patient partner support team. RUG members attended workshops at three key points and were involved in discussion related to the research questions; factors important to patients; findings; and planning for dissemination. Patient partners also reviewed abstracts, presentations and publications, and gave presentations and contributed to discussions at conferences.	Impact of patient partnership included establishing importance of review question, facilitating funding application, identifying additional important factors (leading to amendment of the search strategy and data extraction forms), developing a framework for narrative synthesis based on patient‐identified categories, translating patient concerns into practice recommendations, prioritizing options for dissemination, identifying limitations in the review literature, and informing the next phase of research.	Consult, Involve	Research team
Rhodes et al, (2002), UK	To understand the experience of service users with diabetes who were part of a Users’ Advisory Group for a project evaluating diabetes services in a city in the UK.	Diabetes	A service user advisory group met every 2‐3 months over 2 years and provided input regarding the research process. Group members also participated in the larger steering committee that met twice a year. Feedback about participation was received through taped discussions with advisory group members.	Advisory group members felt that they had impacted the research process as well as had personally gained from the experience of being involved. Advisory group members appreciated being able to connect with other people with diabetes as well as being able to contribute to the community, eg provision of information about services to other people in their community with diabetes. Users contributed to the research process by reviewing research documents (interview guides, surveys) and also gave input regarding new topics for research. The advisory group provided local credibility and access to community networks.	Inform, Consult, Involve, Collaborate	Research team and patient partners
Vale et al, (2012), UK	To evaluate the involvement of patient research partners in the conduct of a systematic review and meta‐analysis from the perspective of patient partners and researchers.	Cervical cancer	Six patient research partners were involved in providing feedback, locating study investigators, interpreting results, contributing to a study newsletter, providing input into a lay summary, and co‐authoring an editorial. Patient partners and researchers completed short surveys with open‐ended questions about their involvement. These responses were coded, and a summary report was sent to all involved. A final meeting was held to discuss the analysis and revise the summary report.	The inclusion of patient partners led to researchers taking on another project related to this topic and an editorial on patient perspectives. There was consensus that patient partners brought a voice that would have been otherwise absent and helped to provide insights into the impact of cervical cancer on women's lives. There was also a sense that the issue of late effects would be explored in future trials given its prominence in the systematic review.	Inform, Consult, Involve	Research team and patient partners
Williamson et al, (2015), UK	To assess the impact of public involvement in the co‐development of an assistive technology for people experiencing foot drop (using functional electrical stimulation (FES)) as a consequence of stroke.	Stroke (with foot drop as a residual side‐effect)	Co‐design process within an assistive technology design study. A lay advisory group of ten people included those with experience with FES, family members, and community members. Activities of the patient partners included activities from conceptualization of the study to end stage dissemination. Advisory group met 9 times. Evaluation of lay advisor experience took place through audio recorded interviews at the beginning, middle and end of the project. A public involvement model based on INVOLVE was used.	The lay advisory group provided input into FES design as well as input into the forthcoming clinical trial of the assistive technology. Participants also reported feeling that they had made a meaningful contribution and a few had also gone on to take part in other research studies as a result of their involvement.	Involve, Collaborate, Lead	Research team
Coser et al, (2014), Canada	To examine the process and personal impact of youth co‐researchers who were involved in a participatory research project about factors that promote resiliency and prevent use of injection drugs for street‐involved youth.	Street‐Involved Youth with Injection Drug Use	Youth with first‐hand experience with street involvement were contracted part‐time for 12 months. Youth co‐researchers were involved with facilitating focus groups, analysis, and dissemination of findings at academic conferences and community meetings, and were paid $15/hour for taking part in the project. Six youth co‐researchers were interviewed at 3 and 7 months into a 12 month project about their experience. Field notes, meeting minutes, and debriefing sessions were also examined.	Youth co‐researchers identified feeling that participation positively influenced their identity, self‐esteem, and sense of meaning for doing work. They felt that they had acquired knowledge and skills that would be transferable beyond the project. Some challenges were related to varying learning abilities of the youth and the need to adapt training and support to accommodate these differences. Researchers noted that they needed to provide both training regarding research as well as support for personal lives given that difficulties that youth had experienced and continued to experience.	Involve, Collaborate, Support	Research team
Revenäs et al, (2018), Sweden	To describe the experiences of stakeholders (people with Parkinson's disease, health‐care professionals, facilitators) with co‐designing an eHealth service intervention.	Parkinson's disease	Four co‐design workshops were held to explore co‐care needs of people with Parkinson's disease and health‐care professionals. Participants included 7 people with Parkinson's disease, 9 health‐care professionals, and 7 facilitators. Participants' feedback on what worked well and what could be done differently was collected on note cards. Facilitators' feedback was provided verbally while a researcher took notes. Researchers also wrote reflections in a diary. After the final workshop, a Web‐based questionnaire was sent to participants to collect data on experiences with the workshops.	Partners contributed their perceived values, challenges, and improvement suggestions. An imbalance in collaboration among stakeholders with diverse backgrounds and expectations was described. Participants desired flexibility and guidance from facilitators. Workshop content was perceived to be relevant, but there were concerns among both the project team and participants about goal achievement. Participants also perceived that co‐design creates hope for future care, but were concerned about health care's readiness for co‐care services.	Participate, Consult, Collaborate	Research team, health‐care professionals, patient partners
Forsythe et al, (2018), USA	To describe patient engagement in PCORI projects and identify the effects of engagement on study design, processes, and outcome selection as reported by PCORI‐funded investigators and partners.	Varied	Patient and other stakeholder research partners answered closed‐ and open‐ended questions through web surveys or phone interviews using the WE‐ENACT tool. Aspects of partner engagement reported include communities represented, study phases in which partners are engaged, engagement approaches used, and partner influence on team dynamics and other research projects.	Partners were engaged across eight possible study phases, from identifying research topics to disseminating results. Outcomes and measurement identification were the most common phase of engagement. Partner engagement influenced the selection of research questions, interventions and outcomes. Partner engagement also contributed to changes to recruitment strategies, enhanced enrolment rates, improved participant retention, more efficient data collection, and more patient‐centred study processes and outcomes. Partners also participated in study conduct (recruitment, data collection, dissemination). More than two‐thirds of investigators indicated that partners had at least a moderate influence.	Consult, Involve	Research team and patient/stakeholder partners
Hertel et al, (2019), USA	To evaluate the impact of patient involvement on the process and outcomes of designing a new primary care clinic service in a large integrated delivery system.	Primary Care settings	Patients contributed to co‐designing a new service in which a lay staff person connects patients with community resources. Twelve patient co‐designers participated in a four‐day design event and eight patient co‐designers participated in a three‐day 'check‐and‐adjust' event 15 months post‐implementation. An interactive orientation session was held prior to the initial design event. Data sources included interviews, event observation and surveys.	Patient partners contributed to a more patient‐centred service design, broader perspective in priority setting, contributed their thoughts and experiences of how intervention would affect patient lives outside of clinic, clarified where service should be located in clinic and contributed diverse community needs that may have been overlooked. Patient partners brought their own expertise and skills to the design activities and described a sense of personal growth (ie learning where to access care, learning new skills).	Consult, Collaborate	Research team and patient partners
McDonald et al (2016), USA	To explore the experiences of scientists and community members within a Community‐Based Participatory Research‐focused project related to violence victimization and health for those with developmental disabilities.	People with disabilities and violence victimization and health	The project included a steering committee which provided leadership (5 scientists and 4 community members with developmental disabilities) and a community advisory board which also included 4 people with developmental disabilities. Interviews and focus groups were conducted to understand participant experiences. Interviews were completed in‐person, over the phone, or written depending on the needs of the participant.	Involved with promoting accessibility and review of project findings. Project team members described developing skills, meeting new people, earning money, and contributing to the study process. Contributes from patient partners improved recruitment and knowledge translation efforts, and enhanced the community‐academic partnership.	Involve, Collaborate	External, independent
Tapp et al, (2017), USA	To examine the impact of patient engagement in a case study of a shared decision‐making study for asthma care	Asthma	Patient partners included those with lived experience who were involved in all aspects of the study, caregiver advocates; research participants; and a patient advisory board. Partners participated in interviews.	Partners contributed to initial project idea development from a patient perspective, suggested changes for simplifying a patient survey, and presented results at research meetings. Caregiver advocates contributed their perspective in study meetings, assisted in data analysis and summarizing themes in study transcripts, advocated for policy changes through membership in wider groups, shared information through personal social media accounts. Research participants trained in research ethics certification, monitored and facilitated study calls between researchers and study sites, advocated for addressing school calendar, flu, and allergy season in asthma visits. Patient advisory board clarified materials for dissemination to patients, contributed to dissemination strategies.	Consult, Involve, and Collaborate	Research team and patient partners

In what follows, we describe data extracted from the included studies in two main areas: (a) contributions of patient partners and related examination of impact; and (b) practical considerations for creating partnerships, including barriers and facilitators influencing patient partnerships. As noted below, the depth of patient involvement varied across studies, with some studies engaging patient partners throughout the research process, and some engaging patient partners for specific tasks. The levels of patient engagement^7^ included in Table 1 highlight these variations. Most studies involved patients in more than one manner (ie Consult, Involve, Collaborate), and slightly more than half of studies demonstrated engagement with patient partners at the stage of ‘Collaboration’.^7^

## 4 PATIENT PARTNER CONTRIBUTIONS

Across studies,^17^, ^18^, ^19^, ^20^, ^21^, ^22^, ^23^, ^24^, ^25^, ^26^, ^27^, ^28^, ^29^, ^30^ patient partner contributions were described with respect to roles enacted and direct involvement in study‐related activities. The impact of partnership was largely reflected in terms of personal experience and gains.

### 4.1 Roles and direct involvement

Patient partners in included studies enacted various roles within the research team. Table 2 provides an overview of these roles.

**Table 2:** Overview of patient partnership roles

	Steering committee membership	Advisory board membership	Consultation	Co‐Design	Knowledge translation	Research tasks
Boaz et al (2016)		X	X	X		
Brown et al (2018)			X		X	
Froggatt et al (2015)	X	X			X	
Howe et al (2017)	X	X				X
Hyde et al (2017)			X		X	X
Rhodes et al (2002)	X				X	
Vale et al (2012)			X		X	X
Williamson et al (2015)		X		X	X	
Coser et al (2014)					X	X
Revenas et al (2018)				X		
Forsythe et al (2018)		X	X		X	X
Hertel et al (2019)	X		X			
McDonald et al (2016)	X	X	X			
Tapp et al (2017)	X	X	X		X	X

Nine included studies described patient partners as members on governance structures within the research study. These governance structures included steering committees^17^, ^19^, ^23^, ^25^, ^29^, ^30^ and advisory boards.^17^, ^18^, ^19^, ^20^, ^25^, ^28^, ^30^ For example, one study^17^ included persons with developmental disabilities on the study steering committee as well as community advisory boards where partners provided input on the development of instruments and tools to assess accessibility for people with developmental disabilities. In two other studies, patient partners acted as advisors during the conduct of a systematic review,^22^ and as leaders in the planning of an annual conference,^23^ respectively. Patient partners in these studies contributed to study outcomes by advancing a research agenda that incorporated patient‐important outcomes.

Eight studies described patient partners enacting consultant‐type roles in the research work.^17^, ^18^, ^22^, ^24^, ^26^, ^28^, ^29^, ^30^ This consulting role varied across studies and included sharing the lived experience of the partner, (eg sharing how the intervention would affect patients^29^) contributing to strategy related to the development of plain language and accessible study materials,^17^, ^24^ and consulting on patient‐centred knowledge translation strategies.^30^

Further, four included studies involved partners as research co‐designers.^18^, ^20^, ^27^, ^29^ For example, in one study, four separate co‐design workshops were organized in order to solicit feedback from partners with Parkinson's disease to inform and explore care needs.^27^ In another study, twelve patient partners participated in a four‐day co‐design workshop with researchers to create a new health‐care service role.^29^ Co‐design activities with patient partners typically involved periods of intense meetings to share ideas and undertake creative activities with other research team members, sometimes followed by ‘check‐ins’ to ensure co‐designed products reflected the contributions of patient partners.^27^, ^29^

In nine of the studies, patient partners were involved in knowledge translation and dissemination efforts in both formal and informal capacities.^19^, ^20^, ^21^, ^22^, ^23^, ^24^, ^26^, ^28^, ^30^ By virtue of being engaged, some patient partners became de facto resources for their communities regarding disease‐specific knowledge.^22^, ^23^ Additionally, and more formally, patient partners presented findings at project meetings^22^ and academic conferences,^19^, ^21^, ^24^ and were engaged in knowledge transfer activities such as networking with various health agencies and academic research partners,^21^ which lent credibility to study results in the eyes of stakeholders.

Six studies discussed patient partners taking on specific research‐related processes and tasks,^21^, ^22^, ^25^, ^26^, ^28^, ^30^ which included the development of research questions,^28^ interview guides and informational materials,^19^ as well as identification of research priorities,^21^, ^28^ and contributions to data analysis^28^ and to regular study briefings.^22^ In one study using a participatory approach, youth co‐researchers interfaced directly with study participants by facilitating focus groups with street‐involved youth.^21^

### 4.2 Impact on personal experience

In addition to the contributions to research processes described above, personal benefits of patient partnership were also noted. For example, patient partners described acquiring practical skills^19^, ^21^, ^25^, ^29^ (eg learning to use a computer^17^) and gaining knowledge about research processes and various topics.^19^, ^21^, ^22^, ^23^, ^29^ Some patient partners reported that the process of collaborating with researchers helped them to gain confidence in identifying themselves as experts and advocates.^17^, ^20^, ^23^ Patient partners also shared that participation in research afforded them a social network of supportive peers; some noted that relationships forged – both personal and professional – lasted well beyond the study period^22^, ^23^ and that participation was a source of positivity (eg laughter)^24^ Finally, patient partners articulated that adding the ‘patient voice’ to research projects and advocating for change was an empowering experience.^19^, ^22^, ^23^ Because of these personal impacts, some studies reported that patient partners sought further opportunities to become involved in research as patient partners beyond study completion.^20^, ^21^

## 5 PRACTICAL CONSIDERATIONS

Practical considerations were addressed largely in terms of barriers and facilitators to patient partnership. A summary of lessons learned, barriers and facilitators to patient partnership, as reported by authors of included studies, can be found in Table 3.

**Table 3:** Barriers and facilitators to patient partner engagement

Author, (year), Country	Reported barriers	Reported facilitators
Boaz et al, (2016), UK	None reported	Dedicating time for patient partnership within an interdisciplinary team Adopting ideas from patient partners to create real change
Brown et al, (2018), UK	Use of jargon, rapid pace of discussions, and focus on ‘academic’ topics during team meetings made it difficult for patient partners to engage Patient partners reported that more time and opportunities to clarify topics of discussion were needed Subtle power dynamics may have inhibited some patient partners from voicing concerns Amount and type of information shared with patient partners was at times overwhelming Academic team members reported that efficiency and productivity of team meetings decreased at times	Strong relationships between academic and patient partner team members increased comfort levels of patient partners Recommendations to facilitate patient partnership include: o Establishing ground rules for clarifying topics within meetings o Creating a glossary of common terms o Providing summaries of long documents o Providing patient partners with necessary skills training, if desired by patient partners o Involving patient partners in ‘core’ research decisions and meetings, and designating other communication and meetings as ‘optional’ to decrease workload and burden on patient partners
Froggatt et al, (2015), UK	Emotional nature of the work caused patient partners experiencing a disease recurrence to resign patient partner roles Use of jargon discouraged participation by patient partners Heavy time commitment for engagement activities was a barrier for some patient partners	Flexibility with respect to time and task commitment of patient partners is required to accommodate changing life circumstances
Howe et al, (2017), UK	Initial tensions around patient partner roles Use of jargon Challenges in ensuring the perspectives of patient partners were heard in meetings	Patient partners had defined project roles and had on‐going support from a lead contact Patient partners were financially compensated and received on‐going training and support Briefing and debriefing patient partners occurred before and after all team meetings Structured opportunities for input by patient partners were threaded throughout the projects Modes and processes of patient partner engagement were flexible and adjusted over time
Hyde et al, (2017), UK	Time pressures for building a patient partnership network, developing relationships, and allowing for multiple points of patient partner involvement Adequate resources (funding and time) to support patient partnership Discontinuity of patient partner involvement as life circumstances change over the course of projects Power imbalances between researcher team and patient partners	Recommendations to facilitate patient partnership include: o Use of an existing network to recruit patient partners o Involvement of patient partners early and often in the research process o Allow for the possibility of an extended study timeline when partnering with patients o Budget for both patient partner compensation and engagement events o Ensure clear expectations and flexibility in patient partner roles o Use small groups to facilitate a relaxed atmosphere o Create different avenues for patient partner contributions (eg online, written, in‐person) o Having a dedicated patient partner lead within a support team
Rhodes et al, (2002), UK	Daytime meetings excluded those with full‐time jobs Use of jargon Patient partners voiced the need for more information and training about the research itself, and how patient partners could contribute Patient partner involvement was time consuming	Personal contact encouraged participation and continued patient partner commitment Valuing of patient partner contributions through reassurance and integration of patient partners into management structure Patient partner meetings in small groups Tailoring tasks to knowledge and expertise of patient partners
Vale et al, (2012), UK	Involving and/or collaborating with patient partners required additional time, effort Aspects of the research process may be upsetting for patient partners Some decisions such as those related to outcomes in a systematic review are ‘pre‐set’ based on that which is collected in the individual studies Difficult to align the needs of patient partners and clinical/scientific collaborators	Recruitment via existing networks (eg through organizations or other volunteers) Using a small group of patient partners Providing information (eg a booklet) upfront that is aligned with patient partner training and support Establishing and maintaining good working relationships among the team including on‐going updates (eg delays in research progress) Communicating the value of patient partners Recommendations to facilitate patient partnership include: o Hosting an information session early in the research process that allows potential patient partners to attend and decide if they wish to be involved
Williamson et al, (2015), UK	None reported	Hosting a workshop to prepare the research team to work with patient partners Use of plain language materials (eg agenda, notes) in font size 14 or larger with visually accessible colours Instructions and expectations clearly described (eg location map) and distributed in advance Offering materials to be distributed on paper or in email and allowing patient partners to select their preference Designating time to clarify questions related to meeting agenda Compensation of patient partners for their time and accommodate needs
Coser et al, (2014), Canada	Need to accommodate and support patient partners with challenging lived experience and trauma (eg youth experiencing poverty, homelessness, and health challenges) Accommodations and support for patient partners can be expensive and time consuming	Supportive relationships between researcher team and patient partners Empowering patient partners to share personal experience External supports (eg youth counsellor) made available
Revenäs et al, (2018), Sweden	Power imbalances between research team and patient partners Differences in knowledge and expectations (eg communication) Time investment and commitment required Perceived readiness of the health‐care system related to co‐care service	Flexibility and ability to time manage Clear description of roles and responsibilities Sufficient space and equipment to support patient partnership
Forsythe et al, (2018), USA	Patient partner health challenges Challenges finding, recruiting, and fully involving diverse patient partners	None reported
Hertel et al, (2019), USA	Expectations regarding behaviour of patient partners (eg future interactions with research team if patient partner had previous negative experience with care) Use of jargon	Including a group of patient partners to enhance representativeness Maintaining a ‘democratic’ atmosphere to decrease power differentials Use of an experience facilitator and small groups for discussion Communicating respect for patient partner contributions Use of electronic health record data permitted more extensive recruitment external to existing networks
McDonald et al (2016), USA	Challenges identifying and upholding accommodation needs for patient partners (eg those that relate to differing abilities) Lack of diversity or representation of range of individuals with disabilities Sharing power among the whole team	Use of value‐based action (eg modelling inclusive partnership from senior leadership) Use of reflexivity Co‐development of policy and structure (eg decision making)
Tapp et al, (2017), USA	None reported	Establishing trusting relationships Compensating patient partners for their time and expertise Using plain language Willingness to clearly explain updates and study progress Inclusion of ‘patient voice’ standing item on all agendas

### 5.1 Barriers

Eleven studies cited specific barriers to partnership encountered during their work.^17^, ^19^, ^21^, ^22^, ^23^, ^24^, ^25^, ^26^, ^27^, ^28^, ^29^ These barriers were varied and encompassed study logistics, team characteristics and perceptions of patient partner roles by researchers, as well as partners themselves. One of the most common barriers identified was the use of jargon.^19^, ^23^, ^24^, ^25^, ^29^ In one study, both researchers and patient partners acknowledged that the nature of some discussion topics, such as prospective funding sources or ethics applications, made it difficult for patient partners to understand and follow what was being discussed.^24^ Researchers in this study reported feeling pressured to limit their conversations to ‘non‐academic’ topics, in order to limit the use of jargon.^24^ Other common barriers described included power imbalances between the researcher and the patient partners,^17^, ^24^, ^26^, ^27^ and the impact of time pressures on the research process.^19^, ^21^, ^22^, ^23^, ^24^, ^26^, ^27^ Other less frequently reported barriers included logistical hurdles, such as meeting disability accommodations,^17^ as well as challenges with retention of patient partners in studies where patient partners experienced changing life circumstances or disease recurrence, limiting their ability to continue their participation in the project.^19^, ^26^ In some instances, partnerships were found to burden both patients and researchers with emotional tolls and burnout. For example, in one study patient partners experienced an emotional burden if confronted with the possibility of a recurrence of their disease while participating in patient partner activities.^19^ With respect to additional challenges, two studies cited financial resource shortage issues,^21^, ^26^ one study referred to fearing patient ‘tokenism’,^17^ and one study indicated group conflict^21^ as key barriers to forging and maintaining partnerships.

### 5.2 Facilitators

Studies also noted several facilitators to engaging in patient partnerships. One common facilitator cited was valuing the patient partner role,^17^, ^22^, ^23^, ^29^ which was accomplished when parties including the research leadership team, team members and patient partners themselves expressed the value and worth of patient partner contributions. Aligned with this facilitator, it was also identified that clear role descriptions and responsibilities for patient partners were important factors for facilitating effective partnerships.^25^, ^26^, ^27^ For patient partners, this often meant the use of explicit policies or guiding documentation to support these descriptions.^25^, ^26^, ^27^ Additionally, studies reported that meeting the personal needs of patient partners (ie disability accommodations, scheduling adjustments, provision of refreshments and transportation) helped to remove barriers to partnerships.^17^, ^20^, ^21^, ^25^, ^26^ Other studies noted the critical role that compensation for time and work played in facilitating patient partnerships.^25^, ^26^, ^30^ An atmosphere of camaraderie between researchers and patient partners, as well as among patient partners themselves,^21^, ^23^ was also reported as facilitative of patient partnership. Additional facilitators noted were purposeful recruitment of patient partners through existing organizations,^22^ sufficient time and space for partnership,^18^, ^24^, ^27^ and flexibility and responsivity (eg personal contact^23^ and preparedness^27^) of the research team.^23^, ^26^, ^27^

### 5.3 Evaluation of patient partnership

In included studies, patient partnership was predominantly evaluated by the research team itself. In four included studies, researchers themselves evaluated the impact of partnership,^20^, ^21^, ^24^, ^26^ and in seven studies, this was jointly evaluated with patient partners or other stakeholders.^19^, ^22^, ^23^, ^27^, ^28^, ^29^, ^30^ Only two of the included studies used an external, independent review process.^17^, ^25^ As this field continues to develop, there may be further opportunity to look to external and independent evaluation of partnership on both outcomes and process. This may reduce the risk for bias, as well as enhance those facilitators related to team cohesion, trust and roles/responsibilities.

## 6 DISCUSSION

Building on the work of previous systematic reviews focused on patient engagement, this scoping review sought to synthesize current available evidence surrounding patient partnership in the research process and to identify impact on research process and research outcomes. Our findings draw attention to the paucity of research where patient partnership is evaluated quantitatively, as all studies included in this scoping review drew on qualitative techniques, with interviews and focus groups primarily used to evaluate partnership strategies. Across studies, teams grappled with the concrete impact of their partnership strategies. Results highlighted that patient partners took on various roles within the research process, and experienced personal impact related to knowledge and skill development, relationship building, and contribution of the ‘patient voice’ to research. Other researchers have noted similar findings in relation to patient engagement research in general (ie not solely focused on patient partners), whereby there is an evaluative focus on the process of patient engagement and its personal impact on patients.^31^, ^32^

There remains pressure for researchers to conceptualize concrete outcomes of patient partnerships to determine the ‘value add’ of involving patients in health research.^12^ Examples of attempts to quantify the benefits of engaging patient partners in research include the development and application of economic equations to theoretically quantify the financial value of engagement,^33^ and the development and testing of measures such as the Patient Engagement in Research Scale (PEIRS)^34^ and Public and Patient Engagement and Evaluation Tool (PPEET),^35^ among others.^36^ The evidence base derived from use of these tools is developing and remains in the early stages. Moreover, evaluation of impact of patient partnership has tended to focus on impact on the research process versus impact on the outcomes of the research.^36^

Quantifying the impact of patient partnerships on research outcomes remains elusive. As such, where patient partnership seems best evaluated may be when it is positioned as a quality indicator around research processes. Patient partnerships represent complex relationships within research teams, which may be enacted through a wide variety of strategies, dependent on the needs of the project, resources available, and the capacity and desire for involvement of patient partners themselves. Manafo, Petermann, Vandall‐Walker and Mason‐Lai^7^ describe varying levels of patient engagement that progress from ‘learning and informing’ to ‘leading and supporting’. Building off of this framework, findings from this scoping review support the need for enhanced guidance and understanding regarding how and what aspects of patient partnership can and should be evaluated. Examples of aspects of patient partnership evaluated in included studies include patient partner training sessions,^18^ patient experiences of research participation,^19^, ^25^ and personal impacts of participation experienced by patient partners.^20^, ^21^, ^23^ Within the extant body of literature on patient engagement, other examples of process and outcome measures for effective engagement exist that could reasonably be extended to evaluation strategies for patient partnership (see Esmail et al^31^).

Existing tools, such as those found within the Centre of Excellence on Partnership with Patients and the Public (CEPPP),^37^ may be of use in the evaluation process. Examples of evaluation strategies for assessing the quality of patient partnerships include use of initial, midpoint and end‐of‐project surveys to understand the evolving relationships between patient partners and researchers, and what behaviours support productive partnerships.^38^ Researchers wishing to report the impact of patient partner involvement in their work may consider doing so using a standardized method such as the GRIPP2 reporting checklist^39^ for reporting patient and public involvement in research. The GRIPP2 reporting checklist prompts researchers to report the specific definition of patient and public involvement within the study, the methods and stages at which they were involved, and outcomes and impact of involvement, among other factors.^39^ Standardization and transparent reporting of patient partner involvement in research is urgently needed in this field, where ambiguity and divergence in definitions of patient partnership, methods for partner involvement, and reporting results related to patient partner involvement remain the norm. By positioning patient partnerships as a research orientation, the focus of patient‐engaged research would be to include the patient voice in all stages of research, in order to facilitate the many benefits of patient partnerships to both health research and patient partners themselves. As others have articulated, there may also be a ‘moral obligation’ to include those impacted by research in its development.^31^

### 6.1 Barriers and facilitators to partnership

In our review, we found that a number of research teams were thoughtful about enfranchising patient partners in the research process. When working with patients as partners in a research team, power dynamics can influence the effectiveness and successfulness of partnership.^40^ Consenting patients to be patient partners positions patients as ‘subjects that contribute’ rather than as equal team members. By omitting the consenting process in partnership, power balances are more evenly distributed between researchers and patients, recognizing that each individual contributes unique skills and expertise to meet research goals. However, omitting consent processes with patient partners did not always serve to completely ameliorate uneven power dynamics in research teams, and several included studies in this review reported that power imbalances remained problematic barriers within their projects. Some literature suggests that unbalanced power dynamics may be mitigated through co‐production of all processes and deliverables between researcher and partner, capitalizing on all assets, expertise, and capabilities to ensure equal and reciprocal partnerships.^41^, ^42^ Researcher and patient partner co‐production can assist to avoid tokenism and power imbalances.^43^

Our search strategy targeted patients who were not consented as study participants as we aimed to ensure that patient partners were represented as fully integrated team members and not research participants who were then called upon to take on ancillary roles. The process of integrating patient partners into the research study team has been utilized and reported in various ways throughout the literature, such as patient partners contributing to research as members of ‘discussion groups’ – whereby patient partner contributions help to steer long‐ or short‐term research decisions, but are not treated as research data^44^; or co‐design team members – whereby ‘users’ and ‘producers’ of health services interventions work in concert to design individual services or interventions.^45^ Arguments for removing the consent process from patient partners participating as members of the study team include a departure from paternalist ideals of consenting patient partners to studies ‘owned’ by researchers,^45^ and shifting the lens of ‘protection’ of patients to a mutually beneficial partnership between those with lived experience and those researching that experience.^45^

While being enfranchised as a research team member clearly has merits, this approach is not without risk. Removing informed consent processes in order to equalize power imbalances has the potential to introduce an element of risk to patient partners, if done without adequate preparation to facilitate informed partnerships. Inadequate preparation for partnership inhibits engagement by both failing to provide the prerequisite background and skills for patients to fully participate and by introducing risk to patient partners via the potential for misinterpretation or misinformation regarding roles and expectations for the partnership. Patient partners who are not adequately informed may approach their roles seeking direct benefits from their participation, particularly in instances where patients seek expertise from researchers or clinicians for personal reasons.

Resource constraints are known to be another barrier to implementing effective partnership strategies.^1^, ^31^, ^32^ Inherently, compensation, or lack thereof, can contribute to power imbalance between researchers and patient partners. When addressing equity among a team, consideration should be made to address whether and how patient partners are to be compensated for the skills and expertise they bring forward. At present, challenges exist regarding how to quantify the worth of lived experience, and what is to be provided on volunteer basis.^46^ However, several resources exist on the topic of remuneration of patient partners in health research, such as guidelines from the CIHR^47^ and PCORI,^48^ as well as public policies such as those from Diabetes Action Canada.^49^ Generally, available guidelines suggest remuneration in scope with the level of involvement of patient partners within the study. To attenuate power imbalances when working with patient partners, we suggest having open conversations with patient partners to identify whether monetary compensation is expected and what can feasibility be provided to ensure all participating parties – researchers and patients alike – feel valued for the experiences and knowledge which they contribute.

Finally, our scoping review revealed one of the most common barriers across studies to be a lack of training for patient partners. To resolve this issue, it is our contention that partnership requires thoughtful preparation of both researchers and patients to create an informed patient partner engagement experience. Aspects of preparation include clear education, role definition and guidance for both researchers and patient partners,^50^ which may serve to maximize patient partner involvement and contributions to research outcomes, rather than limiting patient partners to only providing reflections of their personal experiences.^2^ Manafo et al^51^ identified common barriers to the practice of engagement across the spectrum of research activities, including inadequate preparation of both patients and researchers for engagement. As the Canadian Health Research Roadmap II suggests, transformational research involves the integration of stakeholders, including patients, into the research process.^8^

Brett et al^11^ reported that a lack of preparation for patient partners led to unease and misunderstandings on the part of patient partners as to their roles and expectations. Confusion, conflict and disappointment in not being given medical support for their conditions through research partnerships have also been reported.^11^ In light of this, researchers may want to consider including an explicit discussion with patients prior to partnership to be clear about the fact that their health‐care needs are separate from research involvement, per se. To support informed patient partnership, we endorse the use of formal recruitment and training programmes for patient partners, and the inclusion of guidelines for patient partnership such as those available from the Canadian Stroke Prevention Network's Patient Engagement Resources.^52^ In addition, the Canadian Institutes of Health Research Working Group on Ethics in Patient Engagement in Research has developed a draft document that provides guidance on useful considerations for researchers wanting to engage patient partners in research.^40^ Research teams who are purposeful about patient partner roles, compensation and expectations may be best positioned to mitigate barriers and implement patient partner engagement.

## 7 LIMITATIONS

The literature included in this review largely reflects studies involving adults with chronic, long‐term health conditions as patient partners in research. To this end, only one included study involved youth as patient partners in their work. Thus, findings may not be applicable to researchers seeking to undertake patient partnerships with youth, or outside of the area of chronic conditions.

Secondly, only studies that did not consent patient partners were included in this review. While this team acknowledges that in some instances, partnership and participant roles may overlap, our interest was specifically in those studies evaluating the impact of patient partnership when patient partners were part of the research team ‘only’, and not holding dual roles as a research partner and participant. As such, the considerable patient engagement and patient partnership literature that required patient partners to formally consent to be part of the research team was excluded. While it is likely that this literature would offer further insights regarding impact of patient partnerships, that would be a different focus than the present review.

## 8 CONCLUSION

Patient partnership in research represents an opportunity to leverage the knowledge, skills and experience of not only researchers and clinicians, but also those that research directly impacts – patients and families – to collaborate towards creating impactful and meaningful change in how research is conducted. This scoping review has synthesized the available literature on patient partnership with respect to contributions, as well as barriers and facilitators to partnership. Consideration of the implementation of patient partner preparation strategies to create informed partnerships may help to mitigate unbalanced power dynamics. Finally, the operationalization of patient partnership roles varies across study designs. Guidance is needed for researchers in terms of evaluating patient partnership processes and outcomes according to the level of patient engagement.

## CONFLICT OF INTEREST

The authors declare that there is no conflict of interest.

## Data Availability

The data that support the findings of this study are available from the corresponding author upon reasonable request.

## Appendix 1

### Sample medline search

Database: Epub Ahead of Print, In‐Process & Other Non‐Indexed Citations, Ovid MEDLINE(R) Daily and Ovid MEDLINE(R) <1946 to Present>

Search Strategy:

1. Community‐Based Participatory Research/
2. participatory research*.mp.
3. community based research*.mp.
4. community research*.mp.
5. community empowered research.mp.
6. (community based organi?ation* adj5 research*).mp.
7. ((patient* or stakeholder* or consumer* or communit* or caregiver* or care giver* or carer* or citizen* or guardian* or lay* or client* or public or advocate* or surrogate* or parent* or relative* or spous*) adj3 (contribut* or consult* or engag* or activat* or opinion* or dialog* or partner* or collaborat* or involve* or participat* or advocacy or initiat* or lead or oriented or representative* or driven or instigat*) adj3 (research* or design*)).mp.
8. 1 or 2 or 3 or 4 or 5 or 6 or 7
9. consumer participation/
10. patient participation/
11. community‐institutional relations/
12. 9 or 10 or 11
13. research design/
14. health services research/
15. 13 or 14
16. 12 and 15
17. 8 or 16

## Appendix 2

### Scoping review screening criteria

**Table:**

	Study Selection Criteria
Screening criteria	Does the evidence involve health‐care‐related research? Does the evidence involve patients or their proxies in the research process? Does the evidence report on outcomes used to measurement patient partnership? Is the study design one of the following: systematic review, RCT, cluster RCT, non‐randomized cluster controlled trials, controlled before and after studies, prospective cohort studies, interrupted time series, mixed methods, qualitative? Were patients partners (ie non‐consented members of the research team?)
